# Transcriptome Analysis of Peripheral Blood Mononuclear Cells Reveals Distinct Immune Response in Asymptomatic and Re-Detectable Positive COVID-19 Patients

**DOI:** 10.3389/fimmu.2021.716075

**Published:** 2021-07-29

**Authors:** Jiaqi Zhang, Dongzi Lin, Kui Li, Xiangming Ding, Lin Li, Yuntao Liu, Dongdong Liu, Jing Lin, Xiangyun Teng, Yizhe Li, Ming Liu, Jian Shen, Xiaodan Wang, Dan He, Yaling Shi, Dawei Wang, Jianhua Xu

**Affiliations:** ^1^Department of Laboratory Medicine, Shunde Hospital of Guangzhou University of Chinese Medicine, Foshan, China; ^2^Department of Laboratory Medicine, The Second Affiliated Hospital of Guangzhou University of Chinese Medicine, Guangzhou, China; ^3^Department of Laboratory Medicine, The Fourth People’s Hospital of Foshan, Foshan, China; ^4^Department of Translational Medicine Research Institute, Guangzhou Huayin Medical Laboratory Center, Ltd, Guangzhou, China; ^5^Department of Bioinformatics, Guangzhou Geneseed Biotech Co., Ltd, Guangzhou, China; ^6^Emergency Department, The Second Affiliated Hospital of Guangzhou University of Chinese Medicine, Guangzhou, China; ^7^Department of Clinical Laboratory, The First People’s Hospital of Foshan, Foshan, China; ^8^Department of Laboratory Medicine, Guangzhou Eighth People’s Hospital, Guangzhou Medical University, Guangzhou, China; ^9^Department of Pulmonary and Critical Care Medicine, Shunde Hospital of Guangzhou University of Chinese Medicine, Foshan, China

**Keywords:** COVID-19, SARS-CoV-2, asymptomatic, re-detectable positive, immune response

## Abstract

The existence of asymptomatic and re-detectable positive coronavirus disease 2019 (COVID-19) patients presents the disease control challenges of COVID-19. Most studies on immune responses in COVID-19 have focused on moderately or severely symptomatic patients; however, little is known about the immune response in asymptomatic and re-detectable positive (RP) patients. Here we performed a comprehensive analysis of the transcriptomic profiles of peripheral blood mononuclear cells (PBMCs) from 48 COVID-19 patients which included 8 asymptomatic, 13 symptomatic, 15 recovered and 12 RP patients. The weighted gene co-expression network analysis (WGCNA) identified six co-expression modules, of which the turquoise module was positively correlated with the asymptomatic, symptomatic, and recovered COVID-19 patients. The red module positively correlated with symptomatic patients only and the blue and brown modules positively correlated with the RP patients. The analysis by single sample gene set enrichment analysis (ssGSEA) revealed a lower level of IFN response and complement activation in the asymptomatic patients compared with the symptomatic, indicating a weaker immune response of the PBMCs in the asymptomatic patients. In addition, gene set enrichment analysis (GSEA) analysis showed the enrichment of TNFα/NF-κB and influenza infection in the RP patients compared with the recovered patients, indicating a hyper-inflammatory immune response in the PBMC of RP patients. Thus our findings could extend our understanding of host immune response during the progression of COVID-19 disease and assist clinical management and the immunotherapy development for COVID-19.

## Introduction

Coronavirus disease 2019 (COVID-19) is an infectious disease caused by severe acute respiratory syndrome coronavirus 2 (SARS-CoV-2), which was declared a pandemic by the WHO on 11 March 2020 (1). As of May 1, 2021, 153 million cases have been reported across 188 countries and territories with more than 3.20 million deaths. Disease in most COVID-19 patients is classified as mild; however, about 20% become seriously ill and require hospitalization due to pneumonia (2). The primary symptoms of COVID-19 are listed as fever, dry cough, and shortness of breath but also include other symptoms such as diarrhea, loss of taste and smell, and rashes (1). However, increasing evidence has shown that individuals infected with SARS-CoV-2 can be asymptomatic and simultaneously silent spreaders of SARS-CoV-2 (3, 4). Identification and isolation of asymptomatic patients as early as possible is critical in controlling COVID-19 outbreaks. According to a recent study, four medical workers aged from 30 to 36 years were re-detectable positive (RP) for SARS-CoV-2 within 5–13 days after recovery, suggesting that these four recovered patients may still have been virus carriers (5). All four patients continued to be asymptomatic upon clinical examination, and chest CT findings showed no change from previous images. RP cases have also been reported by several other studies. For example, one report showed that 10.99% of patients (20/182) were SARS-CoV-2 RNA re-positive, and none of them showed any clinical symptomatic recurrence (6). Both asymptomatic and RP patients are receiving more attention because of their potential infectivity.

As there are no effective drugs available at this moment against SARS-CoV-2 and most patients receive symptomatic treatment, it is essential to understand the host–pathogen biology of COVID-19, which will provide the novel insights into more targeted therapeutic approaches for this disease. Over the past few months, studies have reported the characteristics of innate and adaptive immune responses in SARS-CoV-2 infection, which have helped us to understand the potential pathogenesis of COVID-19 (7, 8). Most of these studies focused on the peripheral immune response of symptomatic patients, particularly those with severe COVID-19 (9, 10). A dysfunctional immune response was observed in patients with severe COVID-19, which triggered a cytokine storm causing severe lung and even systemic pathology (11). However, the immunological features and the molecular mechanisms involved in asymptomatic and RP patients still remains elusive. Here we report the transcriptome profiles of peripheral blood mononuclear cells (PBMCs) from COVID-19 patients including asymptomatic, symptomatic, recovered, and RP groups. We performed a comprehensive bioinformatics analysis of these transcriptome profiles using multiple methods including weighted gene co-expression network analysis (WGCNA) (12), single sample gene set enrichment analysis (ssGSEA) (13), and gene set enrichment analysis (GSEA) (14). Our study revealed a weaker peripheral immune response in the asymptomatic group and a hyper-inflammatory immune response in the RP group.

## Materials and Methods

### Patients

This study was reviewed and approved by the ethics committees of the four hospitals including Guangzhou Eighth People’s Hospital, Shunde Hospital of Guangzhou University of Chinese Medicine, Fourth People’s Hospital of Foshan, and First People’s Hospital of Foshan. Written informed consent was obtained from all participants enrolled in this study. The study enrolled 48 patients with confirmed SARS-CoV-2 admitted to the above four hospitals from March through May in 2020 and classified into four groups. The asymptomatic group (8 cases) included patients who did not have self-perceived or clinically recognizable symptoms but were diagnosed as having COVID-19 according to the Protocol for Prevention and Control of COVID-19 (Edition 6) of the National Health Commission of China. According to the Guidelines for the Diagnosis and Treatment of COVID-19 (7th edition), the symptomatic group (13 cases) was defined as patients with evident clinical symptoms, and the recovered group (15 cases) was defined as patients meeting the discharge standards. The RP group (12 cases) was composed of those with re‐positive results of SARS-CoV-2 nucleic acid during the follow-up period after discharge from hospital. The time between the re‐positive results of the PCR test and discharge from hospital ranged from 2 weeks to 1 month. In addition, we enrolled 22 healthy donors from Shunde Hospital of Guangzhou University of Chinese Medicine. All patients had routine laboratory investigations, including complete blood count, liver function tests, blood gas analysis, and coagulation tests. The PBMC samples were collected within 4 days of admission from asymptomatic, symptomatic and RP groups to maintain consistent timing for comparison between groups.

### RNA Extraction and Library Construction

Total RNA was isolated and purified using TRIzol (Life Services Inc., Glendale, CA, USA) following the manufacturer’s procedure. After the quality inspection of Agilent 2100 Bioanalyzer (Agilent Technologies, Santa Clara, CA, USA) and NanoPhotometer^®^ (IMPLEN GmbH, Munich, Germany), poly (A) RNA was purified from 1μg total RNA using VAHTS^®^ mRNA Capture Beads with Oligo (dT) (Vazyme, Nanjing, China) using two rounds of purification. Then the poly(A) RNA was fragmented into small pieces using VAHTS^®^ Universal V6 RNA-seq Library Prep Kit (Vazyme, Nanjing, China) at 94°C for 8 min. Then the cleaved RNA fragments were reverse-transcribed to create the cDNA with reverse transcription reagent, which was next used to synthesize U-labeled second-stranded DNAs. An A-base was then added to the blunt ends of each strand, preparing them for ligation to the indexed adapters. Each adapter contained a T-base overhang for ligating the adapter to the A-tailed fragmented DNA. After the heat-labile UDG enzyme treatment of the U-labeled second-stranded DNAs, size selection was performed with VAHTS^®^ DNA Clean Beads (Vazyme, Nanjing, China). Then the ligated products were e amplified with PCR. Finally, we performed the 2×150bp paired-end sequencing (PE150) on an Illumina Novaseq™ 6000 (Illumina, Inc., San Diego, CA, USA) following the manufacturer’s recommended protocol.

### Data Processing and Analysis

Cutadapt software (version 1.16) was used to remove the reads containing adaptor contamination. Then we prepared CleanData by removing the low quality bases and undetermined bases. The clean reads were mapped to the hg19 UCSC transcript set using Bowtie2 version 2.1.0 and the gene expression level was estimated using RSEM v1.2.15. 24 immunologically relevant gene sets were collected from the ImmPort database and molecular signature database. The detailed lists of the gene sets are shown in the **Supplementary Table 1**. Gene sets were scored using single-sample gene set enrichment (ssGSEA) analysis, as implemented in the GSVA R package. The statistical analyses were performed using the unpaired, two-sided Mann–Whitney U test. All statistical analyses were performed using R software (version 4.0.2).

### Construction of Co-Expression Modules

First, flashClust function was used to carry out cluster analysis of the samples with the appropriate threshold value to detect and remove the outliers. Then, the soft thresholding power β−value was screened during module construction by the pickSoftThreshold function in the WGCNA package. Once the appropriate power value had been determined when the degree of independence was 0.8, the WGCNA algorithm was performed to construct co−expression networks (modules); the minimum module size was set to 30. Co−expression networks or modules were defined as branches of a hierarchical clustering tree, and each module was assigned a unique color label. Subsequently, the information pertaining to the corresponding genes in each module was extracted. The ClusterProfiler R package was used to perform the GO enrichment analysis of the genes in the modules.

### Construction of Module-Trait Relationships

The WGCNA algorithm utilizes module eigengenes (MEs) to assess the potential correlation of gene modules with clinical traits. The module eigengene was defined as the first principal component of the expression matrix for a given module. The module eigengene can be considered an average gene expression level for all genes in each module. The disease conditions of COVID-19 patients were converted into numerical values and a regression analysis was performed between the module eigengene values and the disease conditions of COVID-19 patients.

## Results

### Differences in Immune Cell Abundance Across Disease Conditions

**Figure 1:**
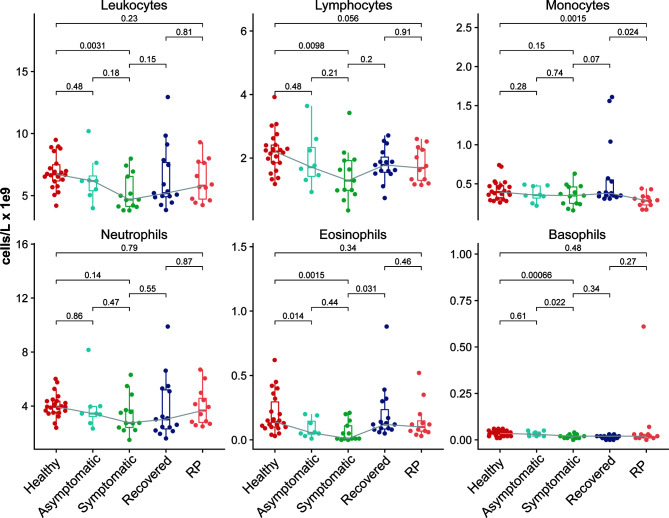
Comparison of immune cell abundance among the COVID-19 patients and healthy donors, with clinical complete blood counts with differential cell count for COVID-19 patients and healthy donors. Each dot represents the value of each individual from the groups (healthy donors, n = 22; asymptomatic, n = 8; symptomatic, n = 13; recovered, n = 15; RP, n=12). The box plots show the medians (middle line) and first and third quartiles (boxes), and the whiskers show 1.5× the IQR above and below the box. Unpaired, two-sided Mann–Whitney U test *p*-values are depicted in the plots.

To investigate the immunological features of asymptomatic and RP patients with COVID-19, we performed mRNA sequencing to study the transcriptomic profiles of PBMCs from 48 COVID-19 patients which include 8 asymptomatic, 13 symptomatic, 15 recovered and 12 RP patients. There were 22 healthy donors included as the control group. The demographic, clinical, and laboratory characteristics of enrolled patients are summarized in **Table 1**. The laboratory results revealed a significant lower level of lymphocytes/leukocytes in symptomatic patients compared with the healthy donors (**Figure 1**), which is consistent with a previous report of lymphocytopenia/leukopenia in COVID-19 patients (2). In addition, eosinophil and basophil counts were also significantly lower than in symptomatic patients compared with the healthy donors, although no significant differences were identified for monocyte and neutrophil counts (**Figure 1**). As shown in **Figure 1**, the lymphocyte counts in asymptomatic patients were higher than in symptomatic patients, although the difference was not significant because of the small sample size. The basophils counts in asymptomatic patients were significantly higher compared with the symptomatic patients. Interestingly, a significantly lower level of monocytes in RP patients was observed when compared with the recovered patients and it was comparable for other blood cell counts between RP and recovered patients.

**Table 1:** Baseline characteristics and laboratory parameters of patients infected with SARS-CoV-2.

	Healthy (n = 22)	Asymptomatic (n = 8)	Symptomatic (n = 13)	Recovered (n = 15)	Re-detectable positive (n = 12)
Age (y, median and IQR)	30.50 (26.75-39.00)	28.50 (21.50-40.75)	33.00 (26.00-48.50)	56.00 (25.00-68.00)	35.00 (25.25-59.75)
Female (n, %)	5 (22.7%)	4 (50%)	11 (84.6%)	5 (33.3%)	6 (50%)
Male (n, %)	17 (77.3%)	4 (50%)	2 (15.4%)	10 (66.7%)	6 (50%)
Signs and symptoms (n, %)					
Cough			5 (38.5%)	3 (20.0%)	1 (8.3%)
Expectoration	0	0	5 (38.5%)	3 (20.0%)	1 (8.3%)
Shortness of breath	0	0	4 (30.8%)	1 (6.7%)	1 (8.3%)
Fatigue	0	0	4 (30.8%)	0 (0)	1 (8.3%)
Sore throat	0	0	3 (23.1%)	0 (0)	1 (8.3%)
Chest tightness	0	0	3 (23.1%)	1 (6.7%)	0 (0)
Dry throat	0	0	3 (23.1%)	0 (0)	1 (8.3%)
Headache	0	0	3 (23.1%)	0 (0)	0 (0)
Chills	0	0	0 (0)	1 (6.7%)	1 (8.3%)
Poor stomach intake	0	0	1 (7.7%)	0 (0)	1 (8.3%)
Gastrointestinal reaction	0	0	0 (0)	0(0)	1 (8.3%)
Palpitation	0	0	0 (0)	0 (0)	1 (8.3%)
Diarrhea	0	0	0 (0)	1 (6.7%)	0 (0)
Myalgia	0	0	0 (0)	1 (6.7%)	0 (0)
Comorbidities (n, %)					
Hypertension	0	0	2 (15.4%)	1 (6.7%)	4 (33.3%)
Diabetes	0	0	2 (15.4%)	1 (6.7%)	2 (16.7%)
Liver disease	0	0	1 (7.7%)	1 (6.7%)	1 (8.3%)
digestive diseases	0	0	1 (7.7%)	1 (6.7%)	0
Kidney disease	0	0	0	1 (6.7%)	1 (8.3%)
Anemia	0	0	0	1 (6.7%)	1 (8.3%)
Cardiovascular disease	0	0	0	1 (6.7%)	0
Thyroid disease	0	1 (12.5%)	0	0	0
Hypertriglyceridemia	0	1 (12.5%)	0	0	0
Rhinitis	0	0	1 (7.7%)	0	0
laboratory parameters (median and IQR)					
Leukocytes (×10^9^/L)	6.71 (6.05-7.83)	6.19 (5.16-7.29)	4.68 (4.11-6.74)	5.20 (4.87-7.86)	5.80 (4.65-7.71)
Lymphocytes (×10^9^/L)	2.21 (1.79-2.46)	1.71 (1.35-2.51)	1.29 (0.92-1.94)	1.78 (1.51-2.18)	1.69 (1.23-2.32)
Monocytes (×10^9^/L)	0.40 (0.31-0.49)	0.36 (0.27-0.48)	0.35 (0.25-0.47)	0.38 (0.34-0.57)	0.29 (0.23-0.34)
Neutrophils (×10^9^/L)	3.95 (3.51-4.40)	3.44 (2.89-3.98)	2.75 (2.32-4.28)	3.02 (2.19-5.30)	3.67 (2.72-4.85)
Eosinophils (×10^9^/L)	0.14 (0.10-0.33)	0.06 (0.04-0.18)	0.01 (0.01-0.13)	0.12 (0.08-0.30)	0.10 (0.06-0.18)
Basophils (×10^9^/L)	0.04 (0.02-0.05)	0.03 (0.02-0.05)	0.02 (0.01-0.03)	0.02 (0.01-0.02)	0.02 (0.01-0.03)

### Identification of Gene Co-Expression Modules

To clarify the critical modules and hub genes that were associated with different status of COVID-19 patients, we performed a weighted gene co-expression network construction analysis (WGCNA) in the 70 samples. Because non-varying genes usually represent noise, we selected the top 3000 varied genes for constructing the weighted gene co-expression network. The cluster analysis by flashClust revealed no outlier samples, and all 70 samples were included in further analysis (**Supplementary Figure 1A**). The power of β was set at 7 to ensure a scale-free network (**Supplementary Figure 1B**). The topological overlap measure (TOM) for each gene pair was then calculated. Hierarchical clustering analysis based on the TOM dissimilarity measure (1-TOM) revealed six modules (**Supplementary Figure 1C**). Each module contained a group of genes with high TOM that were coordinately expressed and potentially involved in similar biological processes. Each module was assigned a unique color to distinguish the individual modules.

### Association of Modules With Disease Conditions

**Figure 2:**
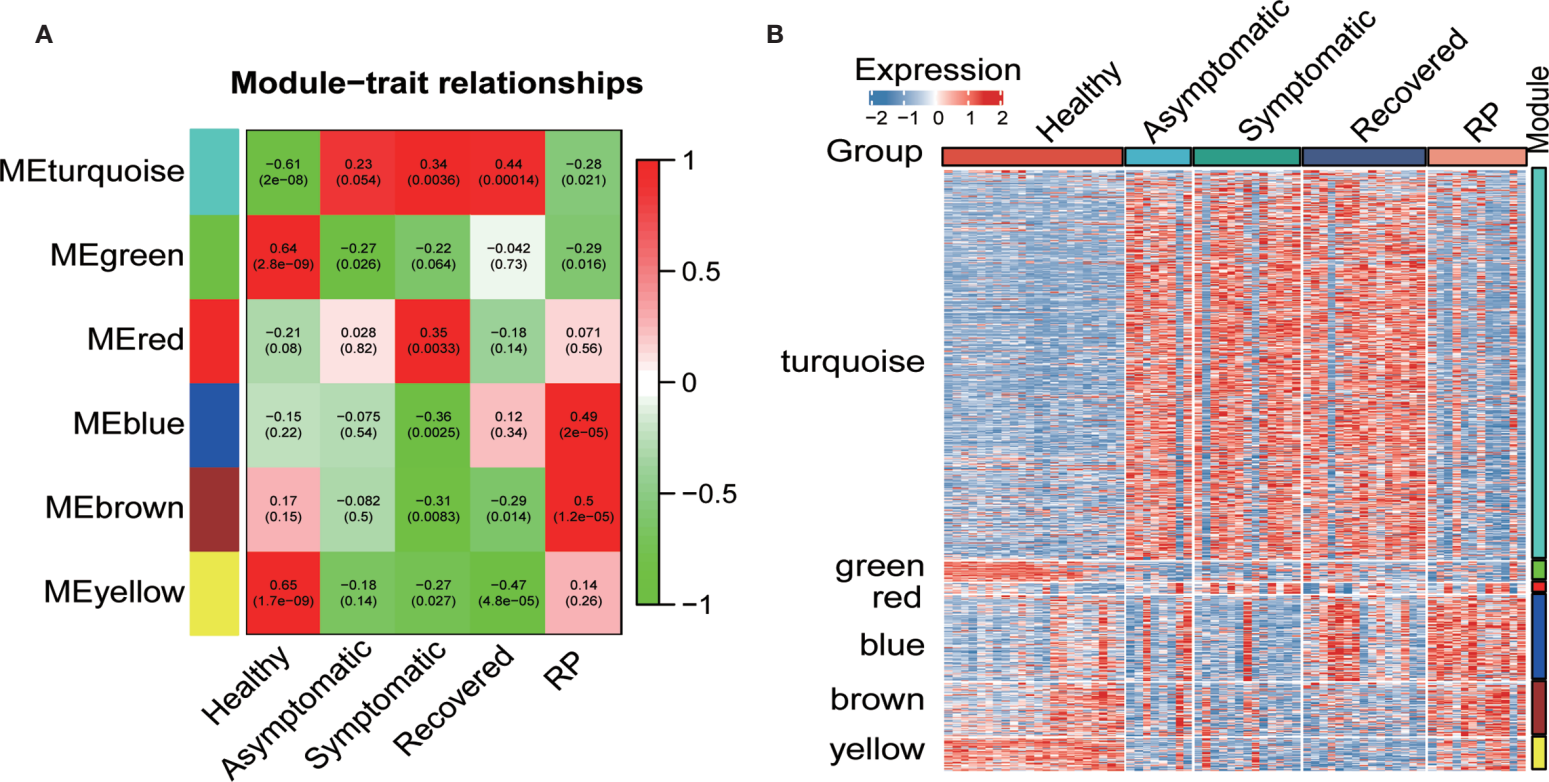
Co-expression network analysis by WGCNA. **(A)** Pearson correlation coefficient matrix among module eigengenes (MEs) and disease conditions in the COVID-19 patients. Each cell reports the correlation (and *p*-value) between module eigengenes (rows) and disease condition (columns). **(B)** Heatmap of the expression of the genes of each module in all samples.

To investigate the association of co−expression modules with the disease conditions of in COVID19 patients, we calculated the correlations between the module eigengenes and the disease conditions in COVID19 patients. As shown in **Figure 2A**, the turquoise module displayed strong positive correlation with the asymptomatic, symptomatic and recovered group, whereas the red module showed a significant positive correlation with the symptomatic group only. Furthermore, both blue and brown modules had a significant positive correlation with the RP group. Consistent with the correlation data, a heatmap revealed higher expression of the turquoise module genes in the asymptomatic, symptomatic and recovered groups and higher expression of the red module genes in the symptomatic group (**Figure 2B**).

### Functional Enrichment Analysis of Genes in Modules of Interest

**Figure 3:**
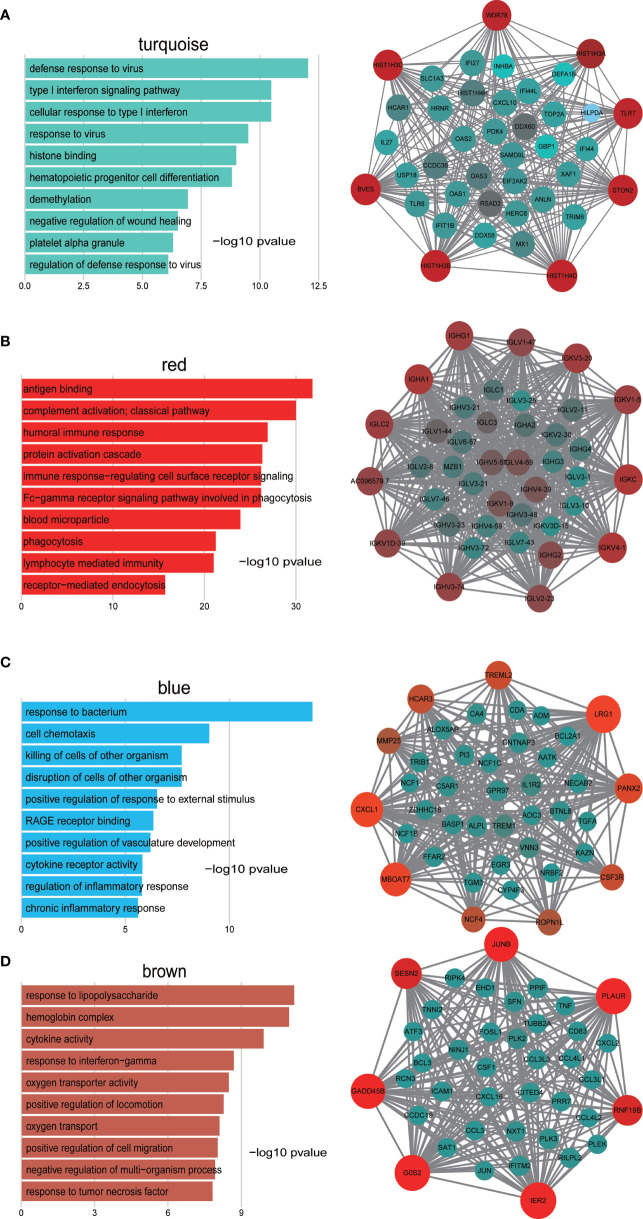
GO enrichment analysis of genes in the turquoise **(A)**, red **(B)**, blue **(C)**, and brown **(D)** modules and visualization of co−expression of genes in the turquoise **(A)**, red **(B)**, blue **(C)**, and brown **(D)** modules. The left panel is the GO enrichment analysis and right panel is the network visualization. The top 40 genes with highest intra-modular connectivity are shown in the network.

To determine the biological function of the four modules of interest, we employed GO enrichment analysis of the genes in each module. As shown in **Figure 3A**, genes in the turquoise module were enriched in response to the virus, response to type I interferon and histone binding. For the red module, the genes were mainly enriched in antigen binding, humoral immune response, and complement activation (**Figure 3B**). Finally, genes in blue module were enriched in response to bacterium and cell chemotaxis and genes in the brown module were enriched in response to lipopolysaccharide, hemoglobin complex and cytokine activity (**Figures 3C, D**).

To investigate in more detail the interplay between genes in the above four modules, we used cytoscape software to visualize the network of targeted modules and the intra-modular connectivity. The top 40 genes with the highest intra-modular connectivity were used for the network visualization. Most of these genes in the turquoise module are related to response to virus and response to type I interferon, which include TLR7, TLR8, CXCL10, IL27, IFI27, IFI44, and TRIM6, et al. (**Figure 3A**). These top genes in the red module are related to humoral immune response, including IGHG1, IGHA1, IGLC2, IGLV1-47, and IGKV3-20, et al. (**Figure 3B**). For the blue module, the top 40 genes include the CXCL1, CSF3R, EGR3, IL1R2, and TREM1, et al, which are related to cell chemotaxis and cytokine receptor activity (**Figure 3C**). For the brown module, the top 40 genes include JUNB, TNF, CXCL2, CXCL3, CCL3, CXCL16, and FOSL1, et al, which are related to response to lipopolysaccharide and response to TNFα (**Figure 3D**).

### Differences in Immune Responses Across Disease Conditions

**Figure 4:**
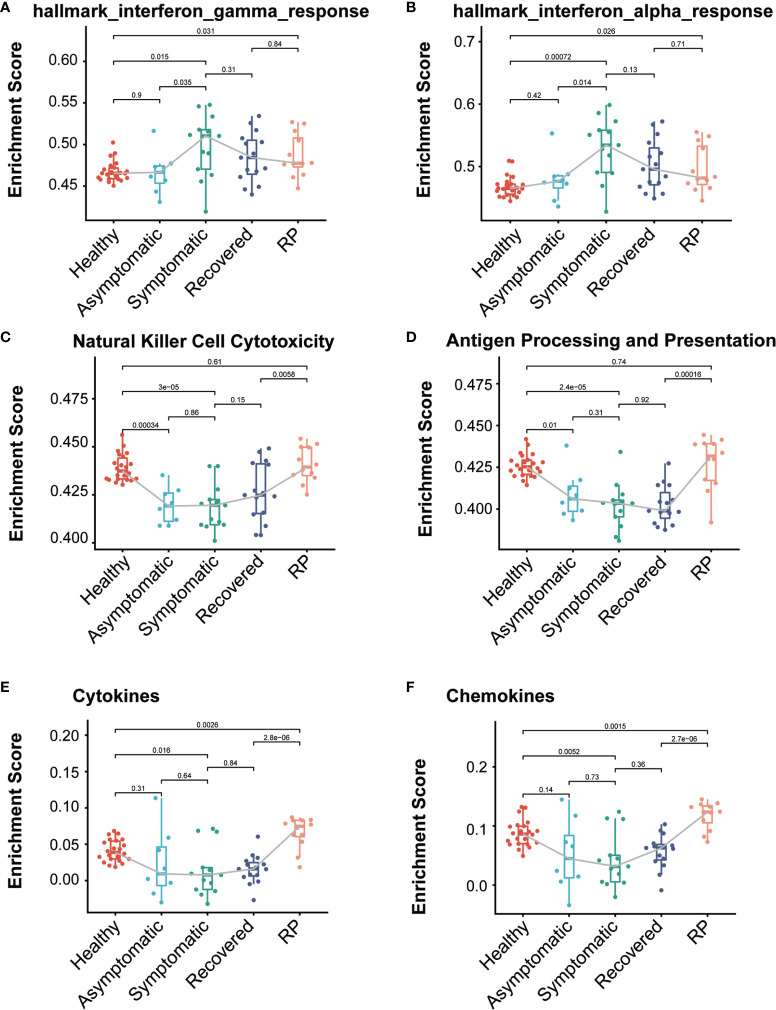
Comparison of immune-relate gene sets activity among the COVID-19 patients and healthy donors. Boxplot of ssGSEA enrichment scores of hallmark_interferon_gamma_response **(A)**, hallmark_interferon_alpha_response **(B)**, Natural Killer Cell Cytotoxicity **(C)**, Antigen Processing and Presentation **(D)**, Cytokines **(E)** and Chemokines **(F)** in individuals from each group (healthy donors, n = 22; asymptomatic, n = 8; symptomatic, n = 13; recovered, n= 15; RP, n=12). The box plots show the medians (middle line) and first and third quartiles (boxes), and the whiskers show 1.5× the IQR above and below the box. Unpaired, two-sided Mann–Whitney U test p values are depicted in the plots, and the significant *p*-value cutoff was set at 0.05.

To identify the difference of immune-related function among these five groups, we collected 24 immunologically relevant gene sets including 17 from the ImmPort database (15) and 7 from the hallmark gene set in the molecular signature database (16). These 24 immune-associated gene sets represented diverse immune functions and pathways (**Supplementary Table 1**). We used the ssGSEA score (13, 17) to quantify the activity or enrichment levels of immune-related functions or pathways in these PBMCs samples. Multiple immune-related functions were dysregulated in PBMCs from COVID-19 patients. The ssGSEA analysis revealed a dynamic change in the interferon response among these five groups. As expected, both the interferon-alpha response and interferon-gamma response activity are significantly higher in the symptomatic group compared with the healthy donors (**Figures 4A, B**). Interestingly, we observed that the levels of interferon response in the asymptomatic group were comparable with those of the healthy group and significantly lower than in the symptomatic group, indicating a lower level of IFN in the serum of asymptomatic patients. Natural killer (NK) cells are an important arm of the innate lymphocytic antiviral response. The level of NK cell cytotoxicity was significantly lower in both asymptomatic and symptomatic patients when compared with the healthy group (**Figure 4C**). The RP patients had a significantly higher level of NK cell cytotoxicity than the recovered patients, indicating a restoration of the dysregulated immune system. In addition, both the symptomatic and asymptomatic patients had a significantly lower level of antigen processing and presentation than the healthy donors, suggesting a defective antigen presentation in the COVID-19 patients (**Figure 4D**). Interestingly, we also observed the RP patients had antigen presentation restored to the normal level. Furthermore, the level of cytokines, chemokines, and interleukins in the PBMCs showed a similar pattern as the antigen processing and presentation (**Figures 4E, F** and **Supplementary Figure 2**). The lower expression of cytokines in symptomatic patients is consistent with previous reports of undetectable expression of most cytokines in the PBMCs of COVID-19 patients (7, 18). The lower level of cytokines in PBMC indicates the elevated serum cytokines largely arise from the local infection site instead of the peripheral blood. On the contrary, the levels of cytokines, chemokines, and interleukins in the RP group are significantly higher than in other groups of COVID-19 patients, indicating a hyper-inflammatory immune response in the PBMC of RP patients.

**Figure 5:**
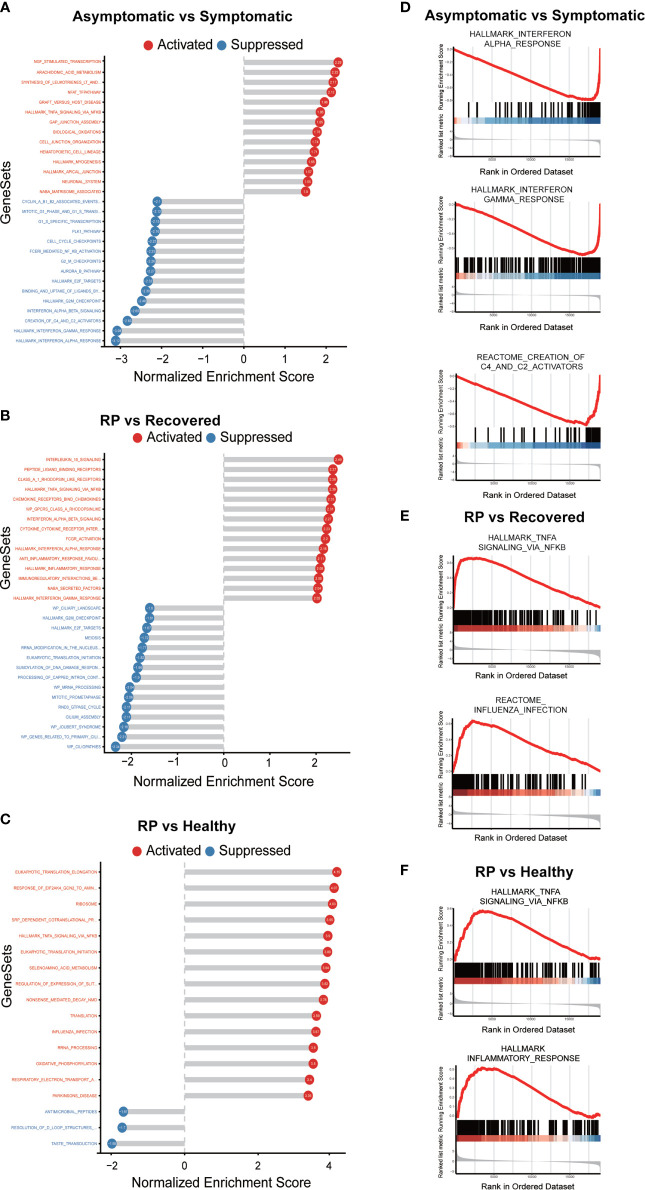
GSEA analysis of the comparison of asymptomatic *vs.* symptomatic, RP *vs.* recovered and RP *vs.* healthy groups. **(A–C)** The top significantly up- and down- enriched gene sets in the comparison of asymptomatic *vs.* symptomatic patients **(A)**, RP *vs.* recovered **(B)** and RP *vs.* healthy groups **(C)**. The status of the gene sets are indicated by red (activation) and blue (suppression). **(D)** Curves of GSEA enrichment scores for the IFN response and complement activators in asymptomatic *vs.* symptomatic group. **(E)** Curves of GSEA enrichment scores for the NF-κB signaling and Influenza infection in RP *vs.* recovered group. **(F)** Curves of GSEA enrichment scores for the NF-κB signaling and inflammatory response in RP *vs.* healthy group.

To further elucidate the up- and down-regulated biological pathways in asymptomatic verse symptomatic, RP verse recovered and RP verse healthy groups, we performed the GSEA analysis (14) to analyze the pathways from KEGG, Reactome and Biocarta as well as the hallmark gene sets from MSigDB. We observed the down-regulation of IFN response and complement activation (“Creation of C4 and C2 activators”) in the asymptomatic patients (**Figures 5A, D**), indicating a weaker immune response of the PBMCs in asymptomatic patients. Interestingly, some cell cycle related pathways were down-regulated and NGF related pathways up-regulated in the asymptomatic patients. The potential role of these pathways needs further investigation. In addition, both TNFα/NF-κB and influenza infection activity were enriched in the RP patients compared with the recovered patients (**Figures 5B, E**). Further, as shown in **Figures 5C, F**, the TNFα/NF-κB and inflammatory response activity were significantly enriched in the RP patients compared with the healthy donors. NF-κB is one of the hallmark signaling factors activated by influenza infection (19). Moreover, a comparative study of COVID-19 and influenza showed the activation of STAT3/NF-κB in PBMCs of patients infected with influenza A virus (IAV)- *vs.* the activation of STAT1/IRF3 in COVID-19 patients (18). Taken together, these findings suggest PBMCs in RP patients have a hyper-inflammatory, flu-like immune response.

## Discussion

COVID-19 patients are characterized by a broad spectrum of disease severity ranging from asymptomatic to critically severe (20). The heterogeneous manifestation of the disease may be due to the patients’ different immune response to SARS-CoV-2. Dysregulated immune responses have been described in symptomatic COVID-19 patients, particularly those with severe disease (9, 21). However, little is known about the immune response in the asymptomatic and RP patients.

Here, we performed the transcriptome analysis on PBMCs from 48 COVID-19 patients in different phases including asymptomatic and RP patients, as well as 22 healthy donors. We found the level of IFN response and complement activation in asymptomatic patients was lower than in the symptomatic patients. This observation is agreement with the data showing lower levels of 18 cytokines including IFN in the serum of the asymptomatic compared with the symptomatic patients (22). It was reported that plasma IFN levels were associated with the COVID-19 disease severity (23) and a recent longitudinal analysis showed that IFNα in peripheral blood was sustained at high levels in patients with severe COVID-19 (24). A robust type I interferon response could exacerbate hyper-inflammation in the progression to severe COVID-19 through diverse mechanisms (25).

WGCNA analysis revealed the genes in the red co-expression module were enriched in the humoral immune response. The red module was found to be positively correlated with the symptomatic patients but not asymptomatic patients, suggesting a high level of humoral immune response in the symptomatic patients. Consistently, two recent studies of serological responses in COVID-19 patients revealed that asymptomatic patients did not respond or have lower antibody levels upon SARS-CoV-2 infection (26, 27). Moreover, the asymptomatic patients were reported to exhibit reduced proportions of SARS-CoV-2 specific T cells, suggesting a weaker cell-mediated immune response (27). Collectively, the well-controlled immune response in the asymptomatic patients may protect the patients from progressing to the inflammatory secondary phase of the disease.

Several recent studies have reported the existence of RP patients and the underlying mechanism of RP occurrence remains unknown (5, 28). The potential reasons might be related to some factors including virology, immunology and sampling methodology. Factors such as false negatives in detection (29), viral residuals (30), intermittent viral release (31) and viral distribution (32) are usually considered major reasons. However, to the best of our knowledge, there is no report on the peripheral immune response in the RP patients. In this study, we identified a hyper-inflammatory immune response in the peripheral blood of RP patients.

Several limitations should be taken into consideration for interpreting this study. First, this study focused on the transcriptome analysis of PBMCs in blood. Adding data on immune cells from lesion sites such as the lung and bronchoalveolar lavage fluid will make the analysis more comprehensive and conclusive. Second, the PBMC samples were collected within 4 days of admission from asymptomatic and symptomatic groups to maintain uniformity of timing for comparison between groups. Future studies with longitudinal samples from COVID-19 patients may help to determine the cause-and-effect relationships between immune response and disease outcome.

In summary, this study will help us extend our understanding of host immune response during the progression of COVID-19 disease, and may help elucidate the COVID-19 infection and provide a basis for rationally designed immuno therapies.

## Data Availability Statement

The datasets generated and analyzed during the current study are available from the corresponding author on request. RNA-seq data will be stored at NCBI/GEO, Accession No. GSE179627.

## Ethics Statement

Written informed consent was obtained from the individual(s) for the publication of any potentially identifiable images or data included in this article.

## Author Contributions

JX, DW, YS, conceptualization, funding acquisition, project administration, resources. YS, DZL, KL, LL, JL, data collection, providing samples. JZ, DDL, XT, YZL, DH, conducting experiments. XW, XD, YTL, ML, JS, data curation, data analysis. JZ, DZL, KL, XD, writing-original draft, writing-review and editing. All authors contributed to the article and approved the submitted version.

## Funding

This study was supported by the Science and Technology Innovation Project of Foshan Municipality (2020001000431) and the National Key Research and Development Project (2020YFA0708001).

## Conflict of Interest

Authors KL, XW and DH were employed by company Guangzhou Huayin Medical Laboratory Center, Ltd. Authors, XD, ML and JS were employed by company Guangzhou Geneseed Biotech Co., Ltd.

The authors declare that the research was conducted in the absence of any commercial or financial relationships that could be construed as a potential conflict of interest.

## Publisher’s Note

All claims expressed in this article are solely those of the authors and do not necessarily represent those of their affiliated organizations, or those of the publisher, the editors and the reviewers. Any product that may be evaluated in this article, or claim that may be made by its manufacturer, is not guaranteed or endorsed by the publisher.
