# Supplementary material for: Transcriptome Analysis of Peripheral Blood Mononuclear Cells Reveals Distinct Immune Response in Asymptomatic and Re-Detectable Positive COVID-19 Patients
Source: Front Immunol. 2021 Jul 29;12:716075. doi: 10.3389/fimmu.2021.716075 (PMC8359015; doi:10.3389/fimmu.2021.716075)
Supplement: Supplementary file 1 [file DataSheet_1.docx]

**Supplementary Figures**


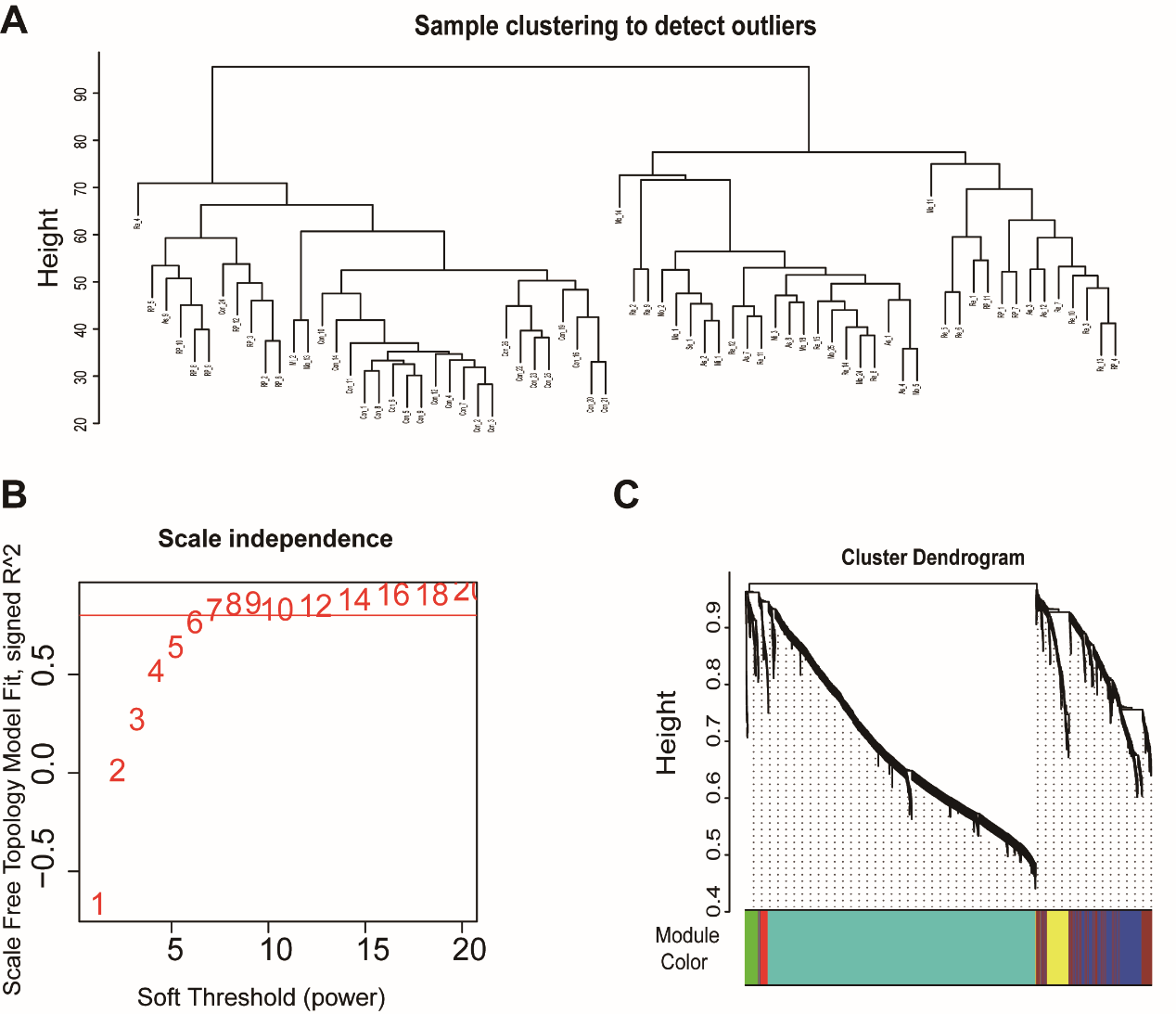


**Supplementary Figure 1**. Co-expression network analysis by WGCNA. (A) Sample clustering for outliers detection. (B) Selection of the soft-thresholding powers. The x-axis is the soft-thresholding power and the y-axis is scale-free fit index. (C) Cluster dendrogram and module assignment from WGCNA. The branches correspond to highly interconnected groups of genes. Colors in the horizontal bar represent the modules.


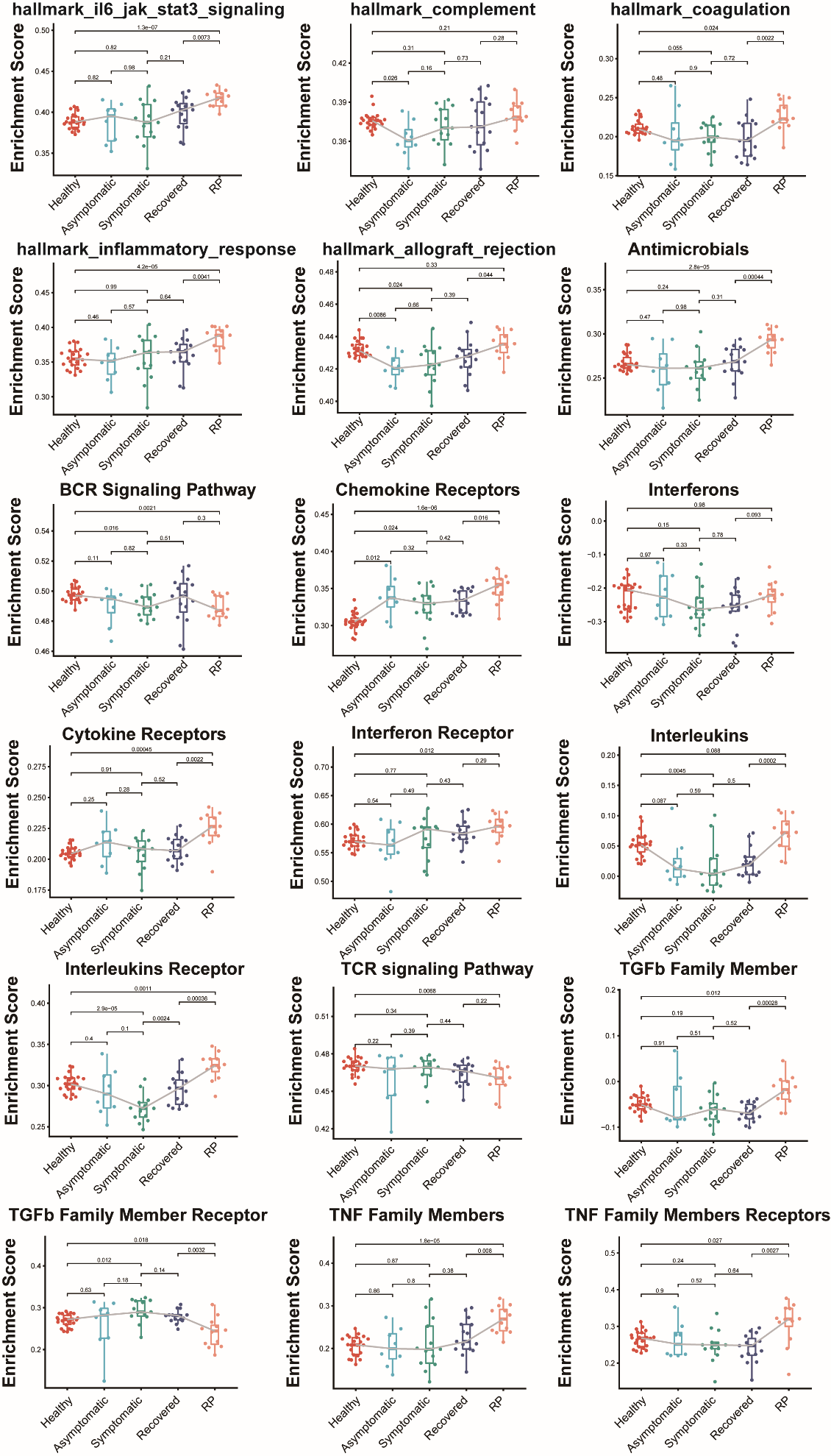


**Supplementary Figure 2**. Comparison of immune-relate gene sets activity among the COVID-19 patients and healthy donors.
